# Sustainable nutrition and the case of vegetable oils to match present and future dietary needs

**DOI:** 10.3389/fpubh.2023.1106083

**Published:** 2023-05-09

**Authors:** Pier Mannuccio Mannucci, Olivier Jolliet, Erik Meijaard, Joanne Slavin, Mario Rasetti, Alberto Aleta, Yamir Moreno, Carlo Agostoni

**Affiliations:** ^1^Fondazione IRCCS Ca' Granda Ospedale Maggiore Policlinico, Angelo Bianchi Bonomi Hemophilia and Thrombosis Center, Milan, Italy; ^2^Department of Environmental Health Sciences, School of Public Health, University of Michigan, Ann Arbor, MI, United States; ^3^Quantitative Sustainability Assessment, DTU-Sustain, Technical University Denmark, Lyngby, Denmark; ^4^Borneo Futures, Bandar Seri Begawan, Brunei; ^5^Department of Food Science and Nutrition, College of Food, Agricultural and Natural Resource Sciences, University of Minnesota, St. Paul, MN, United States; ^6^CENTAI Institute, Torino, Italy; ^7^ISI Foundation, Torino, Italy; ^8^Institute for Biocomputation and Physics of Complex Systems (BIFI), University of Zaragoza, Zaragoza, Spain; ^9^Department of Theoretical Physics, Faculty of Sciences, University of Zaragoza, Zaragoza, Spain; ^10^Pediatric Unit, Fondazione IRCCS Ca' Granda Ospedale Maggiore Policlinico, Milan, Italy; ^11^University of Milan, Milan, Italy

**Keywords:** palm oil, non-communicable disease, cardiovascular disease, diets, sustainable nutrition, saturated fats, complexity science, sustainable development goals

## Abstract

Sustainable nutrition represents a formidable challenge for providing people with healthy, nutritious and affordable food, while reducing waste and impacts on the environment. Acknowledging the complexity and multi-dimensional nature of the food system, this article addresses the main issues related to sustainability in nutrition, existing scientific data and advances in research and related methodologies. Vegetable oils are epitomized as a case study in order to figure out the challenges inherent to sustainable nutrition. Vegetable oils crucially provide people with an affordable source of energy and are essential ingredients of a healthy diet, but entail varying social and environmental costs and benefits. Accordingly, the productive and socioeconomic context encompassing vegetable oils requires interdisciplinary research based on appropriate analyses of big data in populations undergoing emerging behavioral and environmental pressures. Since oils represent a major and growing source of energy at a global level, their role in sustainable nutrition should be considered beyond pure nutritional facts, at the light of soil preservation, local resources and human needs in terms of health, employment and socio-economic development.

## Introduction

Traditional food science is unable to fully define the sustainability of foods in a world where the concept of healthy nutrition is the dominant paradigm in food consumption. The old paradigm arose after the second world war, when conditions of famine were the main concern. Afterwards, the health aspects of foods became the leading concern, particularly in Northern and richer countries, but without considering the emerging need of environmental preservation. Diets that promote adequate nutrition, physical health and environmental sustainability are an aspiration of many (1) and major determinants in the goals of global sustainable development and of the One Health approach (2). Sustainable nutrition encompasses various systems in food production and consumption by simultaneously addressing nutrient adequacy; ecosystem stability; food affordability, availability and safety; waste and loss reduction and sustaining human health in the frame of primary and secondary prevention (3–6).

Because of its complex nature, sustainable nutrition requires a holistic view to interpret all the critical elements along the food chain, from production contexts and impacts to such consequences of consumption as nutrient provision, health benefits and dietary preferences (7). The scientific challenge is to analyze the interactions between these different elements and present them in a way that most consumers can grasp. Moreover, consumers' knowledge of food choices is currently derived from producers, traders, governments and campaigning organizations, each one with their own interests and the final result of consumers' confusion (8). Therefore, the complex concepts of sustainability should be translated into simpler information driving consumers to better evaluate their choices. International exchange of knowledge, with spillovers of agricultural technology and production patterns across countries (9), should ensure an easier access to foods that meet individual consumer needs and health expectations while simultaneously addressing global sustainability and the One Health concept. We have selected the case of vegetable oils, an important source of fat and energy (6), as a relevant example of sustainability in nutrition. A diverse international panel of experts across medicine, nutrition, food science and food environment were assembled to review the nutritional sustainability of vegetable oils.

## The case of vegetable oils

**Figure 1:**
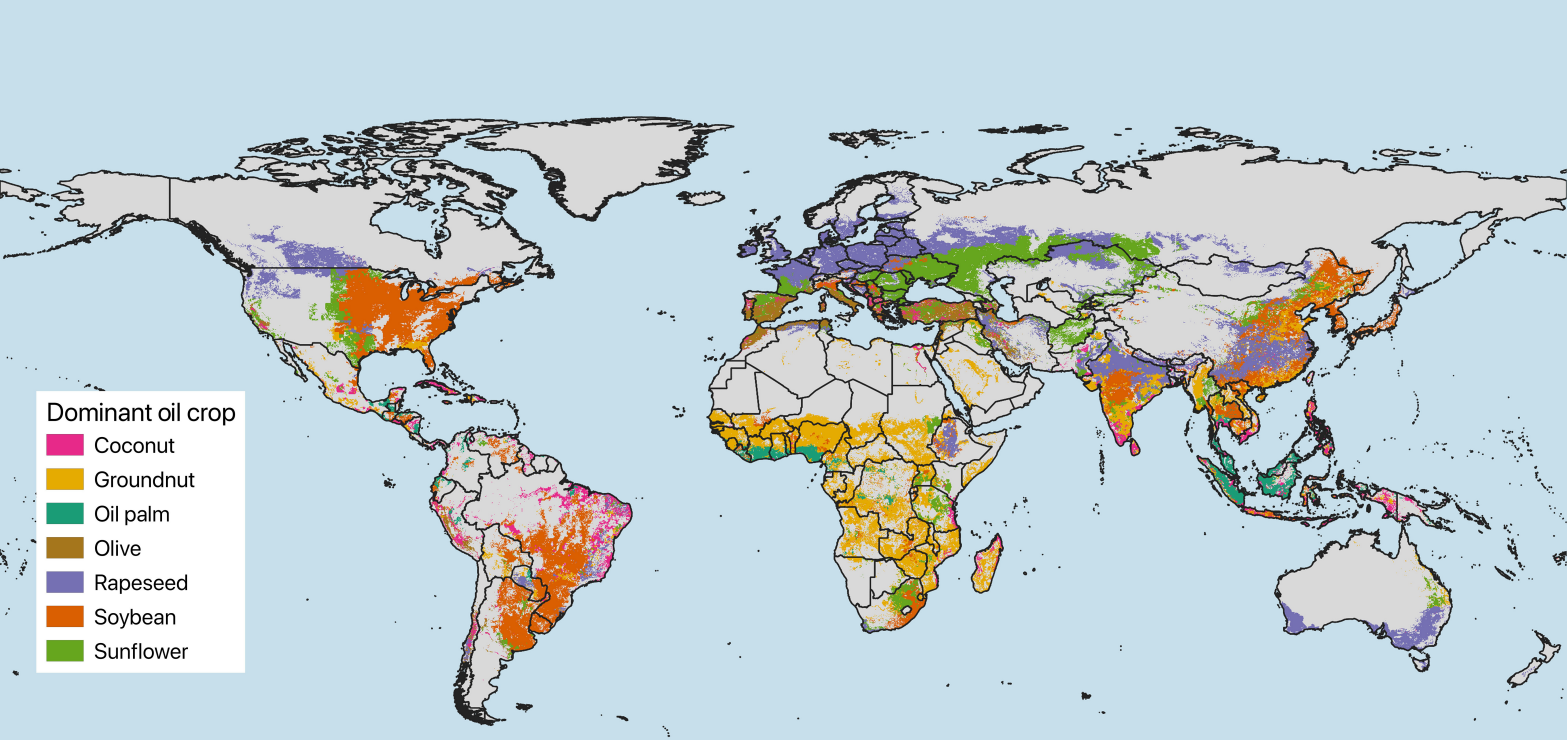
Global map showing the dominant oil crops per grid cell (8). See also text (page 2), for additional information.

Vegetable oils represent a major component of the food system, an important source of energy and an important economic commodity for producers. While in economically advantaged parts of the world there is overconsumption of energy-yielding food components, around 800 million people worldwide were undernourished in 2020 (10). Fats provide 25–30% of daily energy in high income settings (11) and are an affordable food for undernourished people who need increased energy intake (6). Since the 1980s, the global use of vegetable oils has increased across various industrial and consumer segments. The land used for oil crops grew from 114 million hectares (Mha) in 1961 to 332 Mha in 2020 (12), corresponding to ~ 23% of all cropland worldwide (notice that this excludes maize as an oil producing crop). Oil palm, soybean, sunflower seed and rapeseed together account for more than 80% of all the sources of vegetable oil production, with cotton, groundnuts, olive and coconut comprising most of the remainder (8) (Figure 1). Palm oil is principally produced in Indonesia and Malaysia, soy bean oil in South America, sunflower seed oil in Ukraine, Russia, USA and China and rapeseed oil in China, Canada and some European countries (Figure 1). These crops, including soybean (127 Mha planted area) and maize (202 Mha planted area), are also used as animal feed. The global gross production value of oil crops (not including maize) was estimated at US$ 335 billions in 2020 (12). The predicted growth in vegetable oils production because of the rising demand is an environmental concern, because crop expansion has been often associated with tropical deforestation or loss of other natural ecosystems (e.g., woodland savanna, natural grassland) as well as with disruption of biodiversity and other ecosystem values. On the positive side, vegetable oil production can locally lead to higher incomes, generate labor employment and reduce poverty among farms as well as non-farm households (13).

Since the end of the 1980s dietary recommendations have consistently discouraged the intake of animal fats but also of such plant oils rich in saturated fats such as palm and coconut oils (14). The saturated fats supplied by all plant oils and also by other sources can be hydrolyzed to increase their content of polyunsaturated fat, thus leading to the conversion to trans-fatty acids (TFAs) that epidemiological data unequivocally link to an increased risk of cardiovascular disease (15). On the other hand, shorter-chain saturated fats have not been associated with high serum cholesterol levels (6). The 2020 Dietary Guidelines for Americans (DGAs) still support the recommendation of replacing saturated with unsaturated fats, broadly indicating that <10% of total energy should be provided by saturated fat intake but without disentangling the varied effects of saturated fats on serum cholesterol levels. Newer epidemiological data, at odds with these still current recommendations and guidelines on fat consumption, indicate that reducing the intake of saturated fatty acids and replacing them with carbohydrates may be associated as well to adverse effects on blood lipids. By the same token, replacing saturated with unsaturated fats did improve some cardiovascular risk markers but worsened others (16). Furthermore, it must be kept in mind that compounds with potential negative health effects are produced during oil processing, beyond the simple content of saturated fatty acids in vegetable oils (17).

A critical review of the role of dietary saturated fatty acids on cardiovascular disease concluded that the composition of the whole diet is more strongly associated to a lower risk of cardiometabolic disease, than any single nutrient (18). Accordingly, it is being recognized that dietary patterns align not only with healthier outcomes but also with environmental sustainability, consistent with the One Health approach (19). Plant-based dietary patterns commonly defined as healthy—such as the Mediterranean diet, the New Nordic diet, the Japanese diet—translate into different food pyramids but with a shared basis made by vegetables, fruits, whole grains, legumes, nuts and seeds. These diets provide, beyond local sustainability, a wide spectrum of antioxidants with anti-inflammatory properties. Accordingly, healthy dietary pyramids follow indications on vegetable oil consumption inclusive of both tradition and local geographic characteristics, ranging from a relevant role in the Mediterranean diet to a less evident emphasis in the Nordic diet (with milk derived products as a major source of fats) and no clear mention in the Japanese diet, on the whole based on low-fat foods and dressings (e.g., soy). Within these heterogeneous contexts, fats in general and vegetal oils specifically should now be looked at as a complex matrix of multiple nutrients, included in different recipes based on local culinary cultures, and with distinct tradeoffs and synergies among the sustainable development goals of Zero Hunger (SGD 2), Good Health and Wellbeing (SDG 3), and, among others, Life on Earth (SDG 15), Climate Action (SDG 13), and Responsible Production and Consumption (SDG 12) (5). This is the complexity that exemplifies and defines Sustainable Nutrition.

## Environmental and socio-economic impact of vegetable oils

Vegetable oils have been the focus of public media for decades due to controversies on their environmental impacts. In particular, palm oil has been associated with tropical deforestation, often featuring the iconic orangutans (*Pongo* spp.) (20). Other oil crops have generally received less attention, notwithstanding that the total areas allocated to many of them exceed those used for oil palm (8). For example, the oil palm produces ~ 36% of global vegetable oils on 8.6% of the land allocated to oil crops, while soybean produces 26% of oils on 39% of land. Furthermore, the production of groundnut and cottonseed employs relatively large land areas but yields small amounts of oil. Indeed, all oil crops have environmental impacts, ranging from biodiversity impact when their expansion displaces natural ecosystems to water depletion as well as to nitrogen and phosphorus pollution. What is clear is that land requirements vary significantly for different oil crops, with some crops producing much more oil per unit area than others (8). Reducing the land areas allocated to oil production is generally auspicious from an environmental perspective. However, environmental issues are not the only concern pertaining to oil crops, because expansion of crop lands often entails social costs, especially in parts of the world where land or labor rights are weakly defended. On the other hand, there are also benefits, because people living in oil producing areas are able to obtain labor income. Locally produced oils can also provide an affordable source of edible fat to local people, particularly relevant when poor or undernourished (6).

Understanding the impacts of vegetable oil production requires high-resolution and accurate maps showing where these crops are grown. These maps have been produced at a regional scale for soybean, rapeseed and sunflower, but only oil palm and coconut have been accurately mapped at a high resolution and global scale (21, 22). Without these maps it is actually difficult to determine the true impact of the expansion of oil crops on natural ecosystems or other measures of environmental impact (e.g., biodiversity, water pollution, soil health). While palm oil has been associated with tropical deforestation in Indonesia and Malaysia (8) and soybean expansion to loss of forest and woodland savanna in South America (23), environmental impacts have been less well-characterized for other oil crops. There are, for instance, few or no comprehensive data on groundnut and deforestation in Africa, on the water footprint of cottonseed, olive and soybean nor on the relative impacts on biodiversity of pesticides and fertilizers employed for different crops. Similarly, in the frame of a broader sustainable development, there is only limited understanding of how vegetable oil production contributes to different and important development goals (for instance, reducing poverty and hunger, respecting people's rights, preserving the environment). Complex systems and big data analyses might lead to a better understanding on how these issues relate to each other and how they vary under different production systems, i.e., different crops and different scales of production spanning from subsistence-based agroforestry and small-holder plantations to industrial-scale initiatives. Indeed, the production systems may be more relevant for interpreting pathways of sustainability than the individual crops grown in these systems. Vegetable oils are to a significant extent interchangeable (24), and sustainable outcomes are determined more by the production systems than by the oil crops themselves.

In the next few years, the global demand for vegetable oil is expected to further rise alongside a growing human population and related demand for easily available food. Thus, we need to know where oil crops can expand and increase productivity with the least environmental impact. Can this be done by increasing yields on existing lands, or allocating new lands toward oil production? Crops (or indeed production systems) able to better meet a broad range of sustainable development goals should be considered as primary choices. Further research should therefore investigate where crops are grown and how and which are the local synergies and tradeoffs between different socio-economic and environmental goals in the frame of the different production systems. New analytical tools for analyzing these complex scenarios are warranted to clearly visualize and quantify the impact of the different choices for crop production.

## Bridging nutrition and environmental impact

For too long the impacts of nutrition and food consumption have been considered separately from food production processes and related environmental and social impacts. Methods that integrate dietary impacts on human health and natural and socio-economic environments by using consistent and compatible metrics (25) should be developed with the goal to produce health relevant data on the impact of diets, individual foods or food groups such as vegetable oils, as well as to reflect as much as possible the related changes for health rather than just meeting single nutrient recommendations (26, 27). On the environmental side, impacts on a life cycle basis, covering the entire supply chain and including both agricultural production and food processing, should also be assessed. An holistic assessment should also include the role of climate change, land and water use impact, eutrophication on freshwater and marine ecosystems as well as fine particulate matter pollution associated with ammonia emissions.

This assessment effort should be based on the same health metrics used for dietary impacts. Inventory dataset for agriculture processes have recently been developed and are now available for more than 400 food products and ingredients. In particular, the World Food LCA Database and its integration in Ecoinvent (28), the Agribalyse database (29) or the Agri-footprint database (30) cover specific data sets for vegetable oils, including coconut, linseed, maize, olive, palm, peanut, rapeseed, sunflower oil and also margarine. These data enable us to identify the key processes and inputs of these oils on the agricultural production chain. Substantial progress has also addressed the environmental impacts, after comprehensive assessment methods such as ReCiPe or Impact World+ enabled to assess damages on the ecosystem (31). On the nutrition side, the comprehensive Global Burden of Disease (GBD) study systematically analyzed data on nutritional epidemiology, showing that dietary risks play a major role in disease incidence and prevalence and that these risks can be compared by means of the health-based metric DALYs (disability adjusted life *y*ears), already used for other aspects of environmental impact such as for instance particulate air pollution (32, 33). DALYs measures the potential reduction in life expectancy due to early mortality (Years of Life Lost, YLL), as well as the reduction of a healthy life associated with Years Lived with Disability (YLD), expressed using as a disability metric the YLL equivalent. Fifteen risk factors were identified by the GBD study to be associated with nutrients or food groups. Beneficial risk includes milk, nuts and seeds, fruits, fibers and two fatty acid families, i.e., omega-3 fatty acids from seafood and polyunsaturated fatty acids (PUFAs: linoleic acid, LA, and alpha-linolenic acid, ALA) mainly supplied by vegetable oils. GBD risk factors also identified health damage associated with processed meat, red meat, sugar-sweetened beverages (reflected on body mass index), sodium (reflected on blood pressure) and TFAs (33).

**Figure 2:**
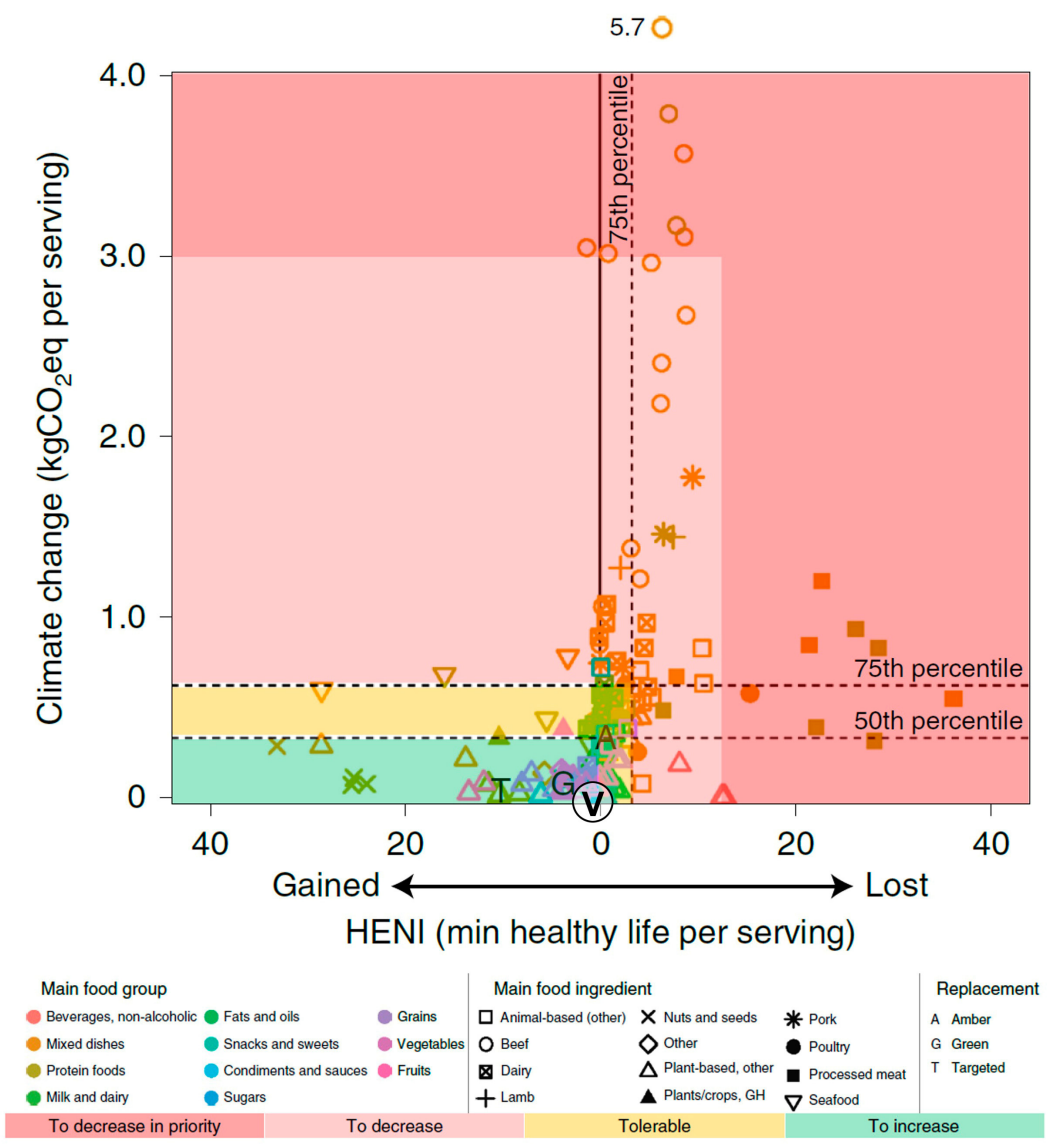
Environmental vs. nutritional impacts for 167 foods representative for the US diet. Nutritional impact of mostly consumed foods (Health Nutrition Index = HENI, *N* = 168) in the US diet as a function of their global warming impacts per serving. Adapted from (34). The circled V denotes average of vegetable oils to the other letters representing consumption-weighted food mixes of the amber-tolerable (A) and green—to increase (G) zones, and a targeted mix of the most nutritionally beneficial foods from both zones (T).

On the whole, nutrition and environmental impacts have been put together in the DALY metric (33, 34), bringing the GBD population-oriented study to the food item level. By analyzing the composition of 5,800 food items in the frame of fifteen GBD risk factors, a Health Nutrition Index (HENI) was produced expressing the impacts of various foods in marginal minutes of life lost or gained within the context of the entire diet. For example, one hot dog leads to 36 min of life lost, whereas a serving of nuts provides 25 additional minutes of healthy life. In comparison, vegetable oils are close to neutral together with grains, fruits and vegetables, between 1 min gained and 2 min lost per serving size (Figure 2).

Comparing the HENI scores with eighteen different environmental metrics that include carbon footprint, water use and air pollution-induced health damages enables us to analyze trade-offs between dietary and environmental performances. Figure 2 shows that, as estimated by the GBD ranking, vegetable oils (identified by V) relative to other foods, both per serving and per kcal, are placed at the low end of carbon and land use while being close to neutrality for dietary impacts on health, together with grains, vegetables and fruits (Figure 2). The Results also suggest that the consumption of beef, pork and lamb meats should be reduced in order to limit the carbon footprint and that processed meat and sweetened sugar beverages should be reduced with high priority from a dietary perspective. What the HENI scores cannot address yet is the relative impacts of different vegetable oils but this should become possible in the near-future.

This modern approach follows the present development of analytical systems that, based on the machine learning approach (artificial intelligence), allow for the extraction of the main effective factors from background noise. For consumers, the environmental impact of food processing and cooking as well as the incidence of these processes on the nutritional quality and nutrient availability should be defined. For vegetable oils more detailed data and information are needed to improve knowledge on the role of individual fatty acids and their combinations and interactions, since current studies often focus on a single dietary factor at a time. Such approaches as survival random forest to large databases containing both dietary intakes of individuals (e.g., European Prospective Investigation into Cancer and Nutrition-EPIC or National Health and Nutrition Examination Survey-NHANES database) and their mortality and morbidity status (e.g., the US National Death Index, NDI) should be employed to address the holistic issue of vegetable oils from crops to consumers with the transversal effects on socio-economical impact, even though it is warranted that data collection occurs over longer time periods (35, 37).

## Next frontiers for innovation and nutrition

Geography originally determined which fats and oils were included in the diet: butter, lard and beef tallow in Northern Europe, North and South America, contrasting with olive, sesame, sunflower seed and soybeans oils in Southern Europe and the rest of the world (35). Unsaturated fatty acid can be converted to a saturated fatty acid by bubbling hydrogen through a heated vegetable oil in a closed vessel. This discovery of hydrogenated vegetable oils, which makes them more solid and spreadable, started an ongoing debate about which fats and oils are “healthy” and which fatty acids are “sustainable”. Adding complexity to the debate on fatty acids is the consideration that fats and oils come from both animal and plant sources.

Previous sections emphasized how data collection is a crucial issue of the path connecting nutrition with health and sustainability within an holistic, complex and multidisciplinary perspective capable to rigorously assess vegetable oils as well as other major dietary components in terms of their impact on health and on such societal and environmental aspects as land allocation to different oil crops and cultivars as well as the impact on the quality of life of local populations and biodiversity preservation. Despite many attempts to homogenize databases, existing data are scarce, heterogeneous and often unreliable (36). Not all the countries (indeed a small fraction of them) provide at least partial food composition tables for their food products and most of them are derived from the analysis of a limited number of samples, a further confounder being the analysis time frame (7). The fat issue needs a fast update, in the frame of complex systems-based views aimed to emphasize novel evidence-based dietary patterns matching sustainability with the goal to overcome the present energetic crisis. To make effective dietary recommendations in order to change food composition and dietary choices, the cultural heritage must also be taken into account, as well as the stability of dietary habits of the same type of food in each country.

## Conclusion

Fats play an important role in the transition to sustainable diets as they are a concentrated energy source that will be needed to future food security (37). Each plant oil can be accessed for its nutritional attributes and ability to be sustainably produced. To accomplish this goal, experts in nutrition, health, agronomy, food production, economy, sociology and governments need to work together with the common goal of feeding the world sustainably in the next future.

There are still knowledge gaps in the concept of sustainable nutrition and how consumers can be provided with metrics that they can easily use for their dietary decision making. Vegetable oils show all the steps of the complex chain connecting agriculture to health across molecular to societal and global environmental scales, including issues such as soil exploitation, climate change impact, role of environmental stressors of the food production processes, health impacts of different foods and the cultural context of nutrition. High resolution data should focus on where crops are grown, needs for fertilizers, environmental impacts and local benefits as indicators of social and economic development. Accordingly collective and global efforts are warranted not only by the scientific community but also by governments, food industry and global health systems in order to build the scientific basis for the introduction of a new paradigm beyond classic dietary patterns, in the frame of a wider holistic approach to individual and global health.

## Author contributions

All authors listed have made a substantial, direct, and intellectual contribution to the work and approved it for publication.
